# Tailored Covalent Organic Frameworks Enable the Encapsulation of Living Cells to Produce Rare Ginsenoside

**DOI:** 10.1002/advs.77136

**Published:** 2026-08-13

**Authors:** Fengmei Liu, Shan Zhang, Shanrong Wei, Ziye Xing, Hongming He, Xiao Liang, Quanshun Li

**Affiliations:** ^1^ School of Life Sciences Key Laboratory for Molecular Enzymology and Engineering of Ministry of Education Jilin University Changchun People's Republic of China; ^2^ College of Chemistry Tianjin Key Laboratory of Structure and Performance for Functional Molecules Academy of Interdisciplinary Studies on Intelligent Molecules Tianjin Normal University Tianjin People's Republic of China

**Keywords:** covalent organic frameworks, encapsulation, rare ginsenoside, whole‐cell catalysis

## Abstract

Whole‐cell catalysis holds great potential in the production of rare ginsenoside compound K (CK), but it is limited by poor mass transfer and inadequate stability in practice. Herein, we report a scalable whole‐cell immobilization platform through in situ assembly of recombinant *Escherichia coli* expressing *Sulfolobus solfataricus* β‐glycosidase with three covalent organic frameworks (COFs: TpPa, TpBD, and TpTAP) in phosphate‐buffered saline. As demonstrated with TpBD, 18.6 grams of TpBD COF could be synthesized in one pot at room‐temperature. The COF shell uniformly encapsulated the cell surface, yielding a robust biocatalyst with high catalytic efficiency, enhanced stability, and excellent recyclability. Specifically, *E. coli*@TpBD‐2 showed 1.98‐fold higher catalytic efficiency than free *E. coli*, while the COF shell significantly improved the *E. coli*’s tolerance to industrially relevant harsh conditions. Notably, the scale‐up synthesis in continuous‐flow reactors using abundant ginsenoside Rb1 as substrate afforded a space‐time yield of 0.67 g·L^−1^·d^−1^ for CK at 70°C, with 75.25% initial conversion rate retained after 10 h of continuous operation. Additionally, this platform enabled the immobilization of various prokaryotic and eukaryotic microbes, demonstrating favorable universality. This work establishes a versatile platform for engineering stable whole‐cell biocatalysts, which is beneficial for facilitating the industrialization of biocatalysis.

## Graphical abstract

This work reports the in situ fabrication of *Escherichia coli*@covalent organic frameworks (*E. coli*@COFs) in phosphate‐buffered saline. The COFs form an exoskeleton on *E. coli*, protecting intracellular β‐glucosidase (Ssbgl) while enhancing mass transfer. In continuous‐flow reactions, the composites efficiently convert ginsenoside Rb1 into rare ginsenoside CK with enhanced catalytic activity, recyclability, and durability.

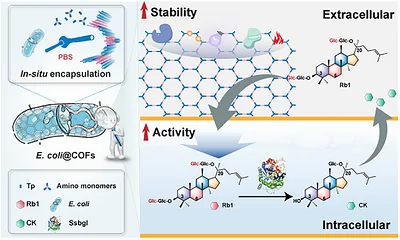

## 1 Introduction

Rare ginsenosides, as secondary metabolites originating from natural prototypical ginsenosides, have emerged as highly promising drug candidates on account of their superior molecular properties, enhanced bioavailability, and potent anti‐cancer and anti‐inflammatory activities [1]. Among them, rare ginsenoside compound K (CK), a deglycosylated metabolite of protopanaxadiol (PPD)‐type saponins, stands out as one of the most pharmacologically active ginsenosides. However, extremely low natural abundance represents a critical bottleneck for its translational development and practical application [2]. Conventional hydrolysis strategies of glycosidic bonds, such as thermal or acid‐base treatments, are characterized by poor selectivity, with side reactions (e.g., hydroxylation and epimerization) leading to unpredictable product distributions [3]. In contrast, enzymatic catalysis offers distinct advantages, including environmental sustainability, high regioselectivity, and mild reaction conditions, thereby positioning it as a promising alternative for CK production [4]. Enzymatic production of rare ginsenosides primarily relies on glycosyltransferases for glycosylation and glycosidases for hydrolysis [5, 6]. Notably, *Sulfolobus solfataricus* β‐glycosidase (Ssbgl) exhibits high efficiency in converting ginsenoside Rb1 to CK via site‐specific cleavage of glycosidic bonds [7]. Nevertheless, free enzymes are plagued by inherent drawbacks, including high preparation costs, cumbersome purification procedures, insufficient industrial stability, and poor reusability. Collectively, these limitations underscore an urgent need for innovative strategies to overcome the inefficiencies of current enzymatic systems in CK biosynthesis.

To address these constraints, synthetic biology‐driven whole‐cell catalysis has been developed, which integrates target enzymes into intact living cells to construct a naturally stable microenvironment for enzymatic activity [8]. This approach not only avoids cumbersome purification of enzymes and lowers the production costs but also preserves the structural and functional integrity of enzymes through the cellular microenvironment. Notably, this strategy has found extensive application in the production of high‐value fine chemicals and the biosynthesis of biofuels [9, 10]. Despite these compelling merits, a key challenge in whole‐cell catalysis stems from the barrier effect of cell walls and membranes, which restricts the transport of substrates and products. Though previous studies have improved the yield of rare ginsenosides by introducing chaperone expression systems or permeabilizing engineered cells [11, 12, 13, 14], such permeabilized cells often undergo significant performance degradation during repeated recovery cycles or upon exposure to environmental fluctuations. Further, the stability of current whole‐cell catalytic systems remains inadequate, which is an indispensable prerequisite for achieving continuous catalytic reactions. Thus, developing a cell engineering strategy that synergistically balances cellular integrity preservation, efficient mass transfer, and robust stability is pivotal for advancing the industrialization of whole‐cell catalysis [15].

Inspired by natural protective mechanisms, functionalized artificial coatings constructed via cell surface engineering have proven to be a promising approach to enhance the stability and functionality of living cells in biocatalytic systems [16, 17]. These coatings endow cells with a controlled microenvironment, which effectively shields them from diverse environmental stresses (e.g., heat, organic solvents, and ultraviolet radiation). Recently, metal‐organic frameworks (MOFs) and covalent organic frameworks (COFs) hold considerable potential for biomacromolecule protection and functional integration, owing to their high porosity, well‐defined open channels, and versatile functionalization capacities [18, 19, 20]. However, due to the high environmental sensitivity of living cells, only a limited number of MOF materials could be synthesized under mild and biocompatible conditions, typically zeolitic imidazolate frameworks, and then used for cell encapsulation [21]. Further, most MOF structures feature narrow pore diameters, polycrystalline architectures, and insufficient stability in diverse environments, thus constraining their utility in cellular protection and functionalization. In contrast, COFs exhibit inherent rigidity derived from strong covalent bonds, enabling them to stabilize biological systems and thereby qualifying as suitable protective exoskeletons for cells [22]. Beyond physical isolation, crystalline COF shells endow the encapsulated cells with robust tolerance against ultraviolet (UV) irradiation, high temperature, extreme pH, and lysozyme [23, 24]. Meanwhile, their precisely tunable mesoporous channels provide the cells with selective, permeable protective barriers, while the highly ordered open pathways within the framework facilitate substrate enrichment and improve mass transfer efficiency [25]. Further, COFs feature fully metal‐free skeletons that confer excellent intrinsic biocompatibility, whereas their rigid covalent networks enable facile separation and repeated recycling in immobilized cell systems [26, 27, 28]. These synergistic advantages collectively render COFs an ideal carrier for constructing artificial exoskeletons for living cells. Unfortunately, traditional COF synthesis requires harsh conditions, including organic solvents, elevated temperatures, and acidic/alkaline catalysts, which significantly hinder their application in biocatalysis [29, 30, 31].

Herein, we report a simple and universal dynamic polymerization strategy using phosphate‐buffered saline (PBS) as the reaction medium, which enables in situ fabrication of COF shells on the surface of *Escherichia coli* in an aqueous, mild, and environmentally benign condition (Scheme 1). This approach achieves rapid and scalable preparation of COF materials. The as‐constructed COF shell integrates the dual functions of a “molecular sieve” and an “artificial exoskeleton.” The precisely tunable channels of the COF shell mitigate the mass transfer limitations for intracellular enzymes, and the highly ordered open frameworks enhance the catalytic activity via substrate enrichment. Moreover, the robust crystalline framework improves the cellular stability under harsh conditions. Additionally, this strategy exhibits broad encapsulation capability toward both eukaryotic and prokaryotic cells. This work establishes a crucial foundation for engineering highly stable and recyclable biocatalysts, thereby offering valuable insights into the sustainable biomanufacturing of rare natural products.

**SCHEME 1:**
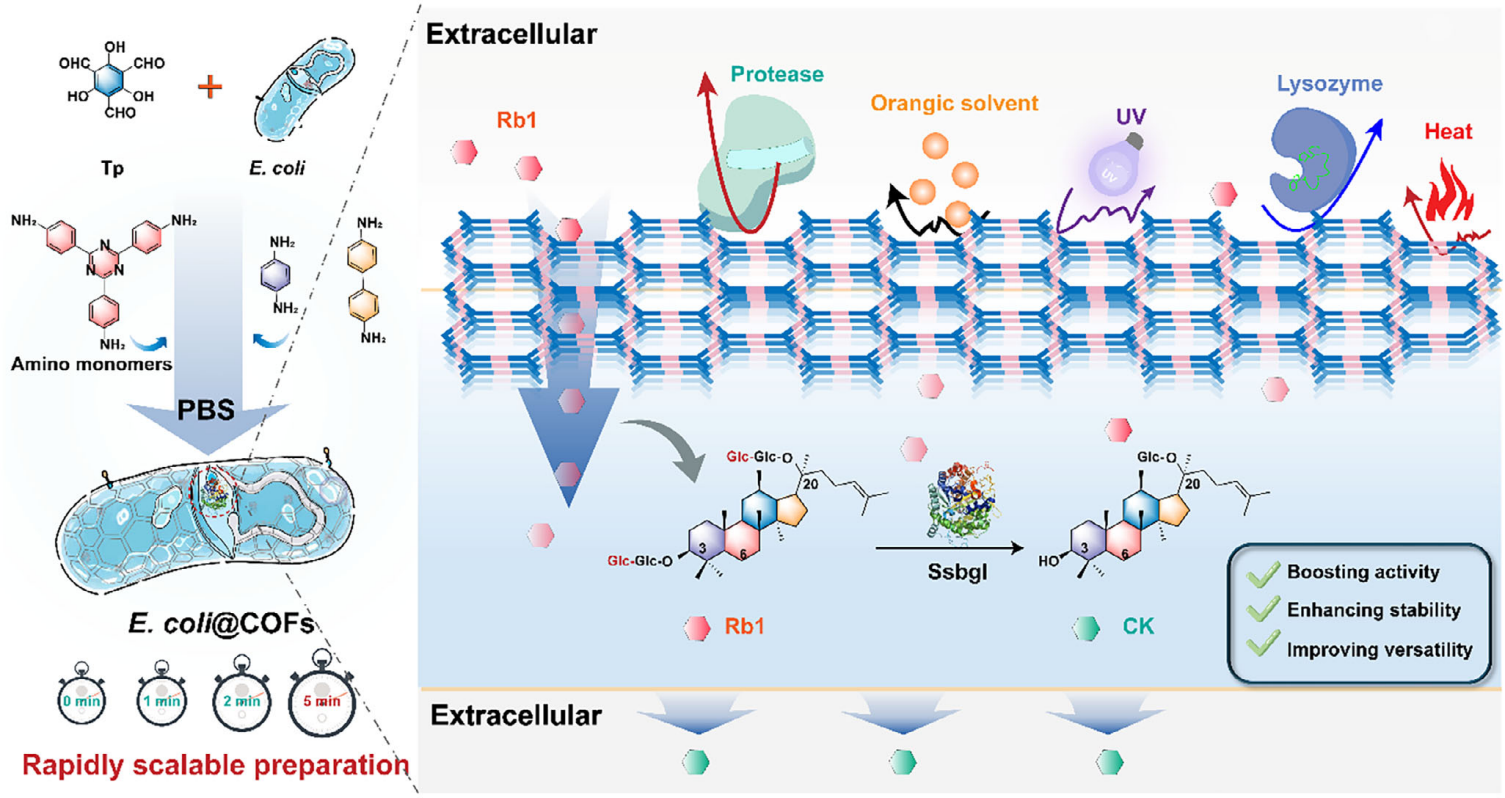
Schematic illustration of the rapid in situ synthesis of *E. coli*@COFs composites using PBS as the reaction medium, highlighting the boosted biological activity and enhanced stability of the encapsulated cells.

## 2 Results and Discussion

### 2.1 Design and Fabrication of a COF‐Based Platform for *E. coli* Immobilization

Traditional COF synthesis mostly relies on organic solvents, but such media often exert adverse effects on cell viability and severely limit their applications in biocatalytic systems [32, 33]. In contrast, a mild buffered system provides a stable and isotonic physiological environment for cells, effectively maintaining cellular activity and functional integrity during the treatment. One of the critical challenges in the aqueous‐phase synthesis of COFs lies in the poor water solubility of organic monomers. Previous studies demonstrated that imidazole could serve as a mild base catalyst to expedite the Schiff‐base condensation and facilitate the construction of β‐ketoenamine‐linked COFs [34]. Accordingly, three kinds of β‐ketoenamine COF materials with distinct pore sizes, designated as TpPa, TpBD, and TpTAP, were prepared using a rapid synthesis approach in PBS (Figure 1a). Solid‐state ^13^C nuclear magnetic resonance (^13^C NMR) spectra displayed characteristic carbon signals corresponding to each material. The multiple peaks in the 100–150 ppm range were attributed to aromatic ring carbons, and the characteristic peak around 184 ppm was assigned to carbonyl carbons (Figure 1b,f,j). These characteristic ^13^C resonances provided direct evidence for the formation of β‐ketoenamine covalent linkages between monomers. Moreover, the synthesis time critically governs the formation of COF frameworks and cell viability. Specifically, an excessively short synthesis period leads to incomplete formation of the COF crystalline framework, whereas prolonged exposure to the synthesis environment results in irreversible impairment of cell viability. Given the dual regulatory role of synthesis time in these two key parameters, we systematically investigated its effects on the crystallinity and porosity of the as‐prepared COFs. Nitrogen adsorption–desorption measurements revealed that the Brunauer–Emmett–Teller (BET) specific surface area of each COF material essentially plateaued at a reaction time of 5 min, with corresponding pore sizes of 1.8, 2.6, and 1.2 nm (Figure 1c,g,k, and Table S1). These pore size values were in agreement with our predefined design targets [35, 36, 37]. Extending the reaction time from 5 to 10 min induced no significant changes in either BET specific surface area or pore size, indicating that the framework structure reached the assembly equilibrium within 5 min. Powder X‐ray diffraction (PXRD) patterns of COFs synthesized at 5 and 10 min exhibited highly analogous peak profiles, which suggested that these COF materials reached assembly equilibrium within 5 min (Figure 1d,h,l). The PXRD patterns of TpPa, TpBD and TpTAP exhibited characteristic diffraction peaks which were matched with the simulated patterns of their corresponding AA‐stacking configurations. All the samples exhibited low‐angle in‐plane (100) reflections and a high‐angle (001) peak derived from the interlayer *π*–*π* stacking, whereas the well‐resolved (200) in‐plane reflection was exclusive to the TpPa sample [38, 39, 40, 41]. The broadened and attenuated diffraction peaks indicated that these materials possessed restricted long‐range crystallinity. This phenomenon was probably originated from the aqueous synthetic route with short reaction time, which greatly hindered crystal growth, generated tiny crystalline domains, and induced interlayer stacking disorder [42]. Fourier transform infrared (FT‐IR) spectra showed distinct characteristic vibrational peaks at 1571^−^ and 1289 cm^−1^ after 5 min of reaction, corresponding to C═C and C─N bonds [43], respectively, further confirming the formation of β‐ketoenamine linkage (Figures S1–S3). Transmission electron microscopy (TEM) and scanning electron microscopy (SEM) images revealed that TpPa, TpBD, and TpTAP exhibited regular spherical structures, two‐dimensional nanosheets, and amorphous aggregates, respectively (Figures S4–S6). High‐resolution TEM (HRTEM) images revealed the local lattice fringes assigned to the (001) planes, with interlayer spacings of approximately 0.34 nm for TpPa, 0.32 nm for TpBD, and 0.34 nm for TpTAP. These values were consistent with the interlayer *π*–*π* stacking distances calculated from the AA‐stacking structural models (Figure 1e,i,m and Figure S7). Notably, this synthesis strategy features simplicity, rapidity, and cost‐effectiveness, endowing it with excellent scalability potential.

**FIGURE 1:**
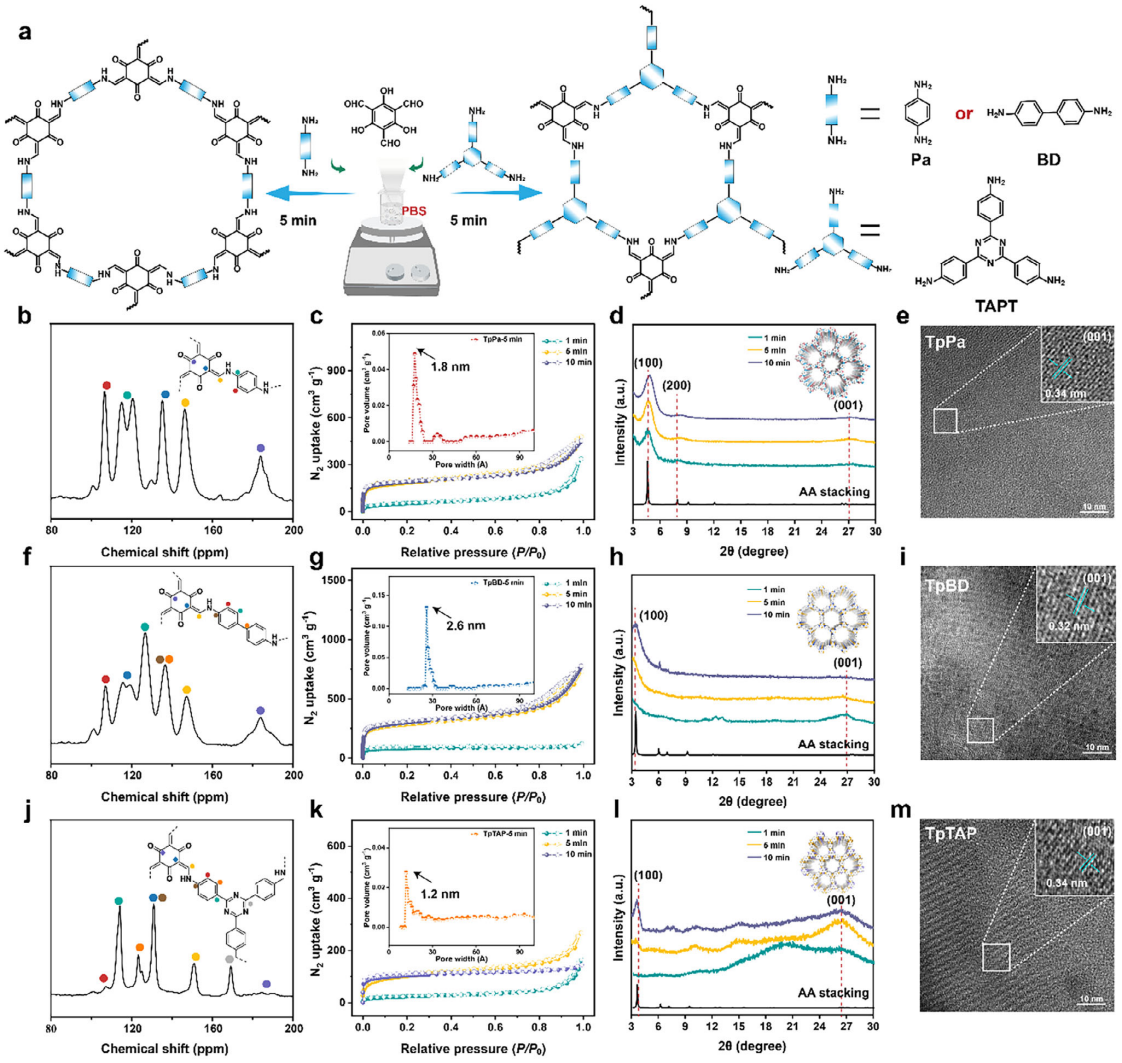
Structural characterization of β‐ketoenamine‐linked COFs for conformational polymer synthesis. (a) Schematic illustration of the synthesis route for conformational polymers linked by β‐ketoenamine‐based COFs. Solid‐state ^13^C NMR spectra (b, f, j), N_2_ adsorption–desorption isotherms with corresponding pore size distributions (insets) (c, g, k), PXRD patterns (d, h, l), and TEM images (e, i, m) of TpPa‐COF (b–e), TpBD‐COF (f–i), and TpTAP‐COF (j–m), respectively.

To validate its feasibility for practical applications, TpBD was selected as a representative for gram‐scale synthesis in PBS [41]. The scaled‐up experiment successfully yielded 18.6 g of TpBD. The BET surface area and pore size distribution analysis revealed that the scaled‐up TpBD‐COF exhibited a specific surface area of 942.65 m^2^/g and a uniform pore width of 2.6 nm (Figure S8a). Moreover, FT‐IR spectra exhibited no shifts in the characteristic vibration of β‐ketoenamine linkage (Figure S8b). Meanwhile, electron microscopy displayed consistent morphological features (Figure S8c,d), and distinct lattice fringes with an interlayer spacing of 0.34 nm were visualized in the corresponding HRTEM images (Figure S8e). Collectively, these results confirmed that our synthesis strategy possessed excellent robustness and reproducibility during scaling‐up, with the resulting materials retaining structural characteristics consistent with those of lab‐scale samples, thereby providing a solid foundation for their practical applications.

In whole‐cell biocatalysis, the cell membrane acts as a physical barrier that impedes mass transfer efficiency between substrates and intracellular Ssbgl, thereby limiting the overall catalytic activity. To tackle this pivotal challenge, we evaluated the effects of physical and chemical permeabilization methods on the catalytic activity of cells [13]. The catalytic efficiency of intracellular Ssbgl was monitored using the hydrolysis of *p*‐nitrophenyl‐β‐D‐glucopyranoside (*p*NPG) to *p*‐nitrophenol (*p*NP) as the readout (Figure S9). The results showed that the treatments with different concentrations of chemical permeabilizers significantly enhanced the catalytic activity of recombinant *E. coli* harboring Ssbgl (Figures S10 and S11). Among them, 5% (v/v) methanol, 0.4 M NaCl, and 0.1% sodium dodecyl sulfate (SDS) exerted the most prominent enhancing effects. Notably, the ‐80°C freezing treatment exhibited favorable permeabilization performance, yielding a catalytic activity of approximately 20‐fold higher than that of intact cells. This remarkable improvement was probably attributed to the “mechanical perforation” effect induced by the formation of ice crystal during freezing, which disrupted the cell membrane. Additionally, low‐temperature conditions triggered the phase transition of membrane lipids, leading to increased membrane fluidity that further facilitated the mass transfer. Further, SEM was employed to characterize the morphological changes of *E. coli* following different permeabilization treatments. As shown in Figure S12, methanol and SDS treatments caused moderate deformation of the overall cell morphology. In contrast, the ‐80°C freezing treatment preserved a cell morphology analogous to that of native *E. coli*, with intact and smooth membrane surfaces. Although some chemical permeabilizers exhibited stronger catalytic performance, they might induce irreversible damage to either cell structure or enzymatic activity. In comparison, the ‐80°C freezing strategy enhanced the membrane permeability to facilitate the mass transfer of substrates while maximally preserving the structural and functional integrity of cells and enzymes. Therefore, the ‐80°C freezing treatment was ultimately selected as the optimal cell permeabilization method for subsequent whole‐cell biocatalysis.

COFs, featuring inherent rigidity from robust covalent linkages, precisely tailorable mesoporous channels, large specific surface areas, and extensive structural diversity, exhibit unique advantages in catalysis and thus could serve as ideal carriers to construct artificial cellular exoskeletons. Three COF‐based protective shells with distinct pore sizes were fabricated on the surface of *E. coli* BL21 cells in PBS within a mere 5 min, denoted as *E. coli*@TpPa, *E. coli*@TpBD, and *E. coli*@TpTAP (Figure 2a). PXRD patterns revealed that the characteristic diffraction peaks of these composites were highly consistent with those of the pristine COFs, confirming that the intact crystalline structure of COF shells was well‐preserved after the cell encapsulation (Figure 2b). Nitrogen adsorption–desorption isotherms demonstrated that the BET specific surface areas of *E. coli*@COFs were significantly lower than those of pure COFs, which was primarily attributed to the internal encapsulation of cellular components into COFs. Meanwhile, their pore size distributions were essentially consistent with those of the original COFs, implying the structural integrity of porous frameworks (Figure 2c, Figure S13, and Table S2). FT‐IR spectra showed no shifts in the characteristic peaks of β‐ketoenamine linkages, indicating that the chemical structure of COFs remained unaltered during the immobilization (Figure 2d). Meanwhile, all the *E. coli*@COF composites exhibited a prominent broad band at 3200–3400 cm^−1^, attributed to the stretching vibrations of N─H moieties from bacterial proteins and O─H moieties from cellular polysaccharides. Further, multiple additional weak signals detected in the 1000–1500 cm^−1^ region were probably originated from various oxygen‐ and nitrogen‐rich functional groups introduced by the encapsulation of *E. coli*. To further investigate the influence of the thickness of COF coating on cellular properties, these three COF‐based composites were each prepared with two thickness gradients (higher monomer feed: ‐1; lower monomer feed: ‐2) by adjusting the monomer dosage, denoted as *E. coli*@TpPa‐1/‐2, *E. coli*@TpBD‐1/‐2, and *E. coli*@TpTAP‐1/‐2. TEM and SEM images clearly showed that the surface of encapsulated cells became roughened and was tightly wrapped by a continuous and uniform COF shell (Figure 2e–h, Figures S14 and S15). Notably, the COF shell thicknesses of *E. coli*@TpPa‐1/‐2, *E. coli*@TpBD‐1/‐2, and *E. coli*@TpTAP‐1/‐2 were measured to be 50.08, 23.87, 43.24, 26.38, 89.04 and 39.66 nm, respectively (Figure S16). These results revealed that the high‐monomer‐feed group exhibited significantly thicker coatings compared to the low‐monomer‐feed group.

**FIGURE 2:**
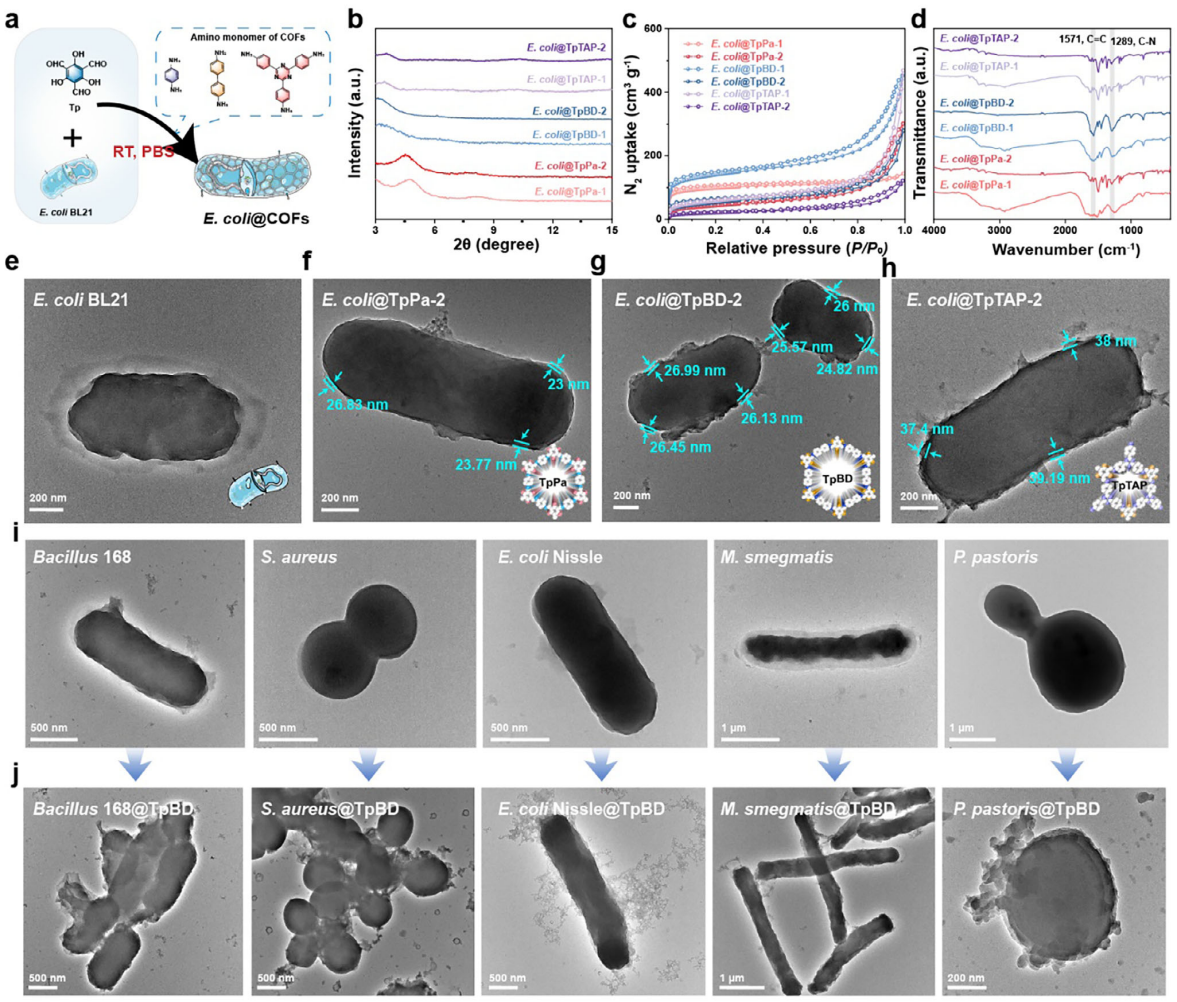
Preparation and structural characterization of *E. coli*@COF composites. (a) Schematic illustration of the preparation process for *E. coli*@COF composites. (b) PXRD patterns, (c) N_2_ adsorption–desorption isotherms, and (d) FT‐IR spectra of *E. coli*@COF composites. TEM images of (e) native *E. coli*, (f) *E. coli*@TpPa‐2, (g) *E. coli*@TpBD‐2, and (h) *E. coli*@TpTAP‐2. The annotated values represent the local COF shell thickness at the marked spots. TEM images of different types of (i) native cells, and (j) TpBD‐coated cells.

To evaluate the biocompatibility of COF‐encapsulated cells, a live/dead cell staining assay was performed [44]. The results demonstrated that the live‐to‐dead cell ratio (green: red) of *E. coli*@COFs was comparable to that of free *E. coli* (Figure S17). Additionally, the biocompatibility was quantitatively analyzed using a multi‐mode fluorescence microplate reader. As presented in Figure S18, all the experimental groups exhibited prominent DMAO fluorescence signals, with weak PI‐derived signals. These results indicated that the encapsulation within COF frameworks did not induce bacterial mortality, implying the excellent biocompatibility of COF matrices for immobilizing *E. coli*. Moreover, when cultured in Luria‐Bertani (LB) liquid medium, *E. coli*@COFs displayed a moderately prolonged exponential growth phase (approximately 8 h delay) relative to native *E. coli* (Figure S19), yet retaining proliferative viability comparable to that of free cells [45, 46]. Collectively, these findings corroborated that the COF‐mediated encapsulation of *E. coli* is a sufficiently mild process, preserving both cellular viability and proliferative potential. To further explore the generalizability of this immobilization strategy, we validated this strategy with five distinct microbial species, including *Bacillus 168*, *Staphylococcus aureus*, *E. coli* Nissle, *Mycobacterium smegmatis*, and *Pichia pastoris*, using TpBD as a representative model. TEM and confocal laser scanning microscopy (CLSM) images confirmed the successful immobilization of all microbial strains (Figure 2i,j, Figures S20 and S21). PXRD and FT‐IR analyses verified that the crystalline structure and chemical bonding of TpBD remained intact across all encapsulation systems (Figure S22). Taken together, these findings demonstrated the broad applicability of this strategy for the surface engineering of diverse microbial species, providing a solid foundation for the development of high‐performance cell‐material hybrid systems.

### 2.2 Enhanced Catalytic Activity of *E. coli*@COFs and Mechanistic Analysis

Subsequently, we systematically evaluated the biocatalytic performance of *E. coli*@COFs. The catalytic activity was determined by monitoring the increase in absorbance at 405 nm, which was caused by the release of *p*NP using *p*NPG as the substrate [7]. Prior to assessing the composite catalysts, we first determined the optimal reaction conditions for free *E. coli* by monitoring its catalytic performance across a range of temperatures and pH values. As shown in Figure S23, both free *E. coli* and purified Ssbgl exhibited optimal catalytic activity at 90°C and pH 7.0, respectively. Further, we determined the activity of different *E. coli*@COFs. The results demonstrated that all the *E. coli*@COFs displayed significantly enhanced activity compared to the uncoated cells, with *E. coli*@TpBD‐1 and *E. coli*@TpBD‐2 exhibiting the highest relative activities with 1.76‐ and 1.98‐fold of that of uncoated cells, respectively (Figure 3a). Notably, the divergent catalytic activity of *E. coli*@TpBD‐1 and *E. coli*@TpBD‐2 was probably caused by the differences in their COF shell thickness. The thin coating afforded abundant substrate‐binding sites while minimizing the diffusion resistance [47, 48]. The thicker coating provided extra adsorption sites but extended the molecular diffusion distance across the framework. The resulting strong mass‐transfer resistance neutralized the benefits of substrate enrichment, lowering the apparent activity [49]. These findings elucidated that the optimal biocatalytic performance was dependent on the balanced interplay of substrate enrichment and molecular diffusion kinetics. To elucidate the mechanism underlying this activity enhancement, we first analyzed the substrate enrichment capacity of the COF‐based composites. As shown in Figure 3b, *E. coli*@TpBD‐1 and *E. coli*@TpBD‐2 exhibited the strongest substrate enrichment ability, and this trend was well‐correlated with their catalytic activity profiles. Moreover, the time‐dependent adsorption capacity (*q_t_*) was recorded to analyze the adsorption kinetics of various *E. coli*@COFs, and the acquired *q_t_*‐time curves were fitted via the pseudo‐second‐order kinetic model [50, 51]. With the elongation of contact time, the adsorption capacity of all *E. coli*@COF composites increased steadily, as the vacant adsorption sites continuously trapped the target molecules (Figure S24). Importantly, nonlinear regression delivered excellent goodness‐of‐fit with R^2^> 0.92 for every sample, which demonstrated that this model could accurately describe the entire adsorption process. Notably, *E. coli*@TpBD‐2 reached the highest adsorption of approximately 40 mg/g at 90 min, far exceeding all other *E. coli*@COF groups. These results indicated that the enhanced catalytic performance was primarily ascribed to the efficient substrate enrichment capability of the COF shell, which not only improved the mass transfer efficiency but also increased the contact frequency between substrates and the active sites of intracellular enzymes [52]. To explore the detailed factors governing the differences in substrate enrichment, we systematically characterized the COF components in terms of pore size, BET specific surface area, hydrophobic interactions, and hydrogen‐bonding interactions (Figure 3c). Pore size and BET specific surface area analyses revealed that both *E. coli*@TpBD‐1 and *E. coli*@TpBD‐2 possessed the largest pore size and the highest specific surface area (Figure 3d). These structural features not only enabled efficient mass transport of substrates and products but also enhanced the local substrate concentration through confinement effects, establishing a dynamic enrichment‐reaction‐release equilibrium system. Notably, despite *E. coli*@TpTAP featuring smaller pore sizes and lower BET specific surface areas than *E. coli*@TpPa, its catalytic activity was significantly higher. This finding indicated that pore size and BET specific surface area were not the sole determinants of catalytic performance [53, 54]. To further clarify additional contributing factors, hydrophobicity analysis was conducted. The results demonstrated that *E. coli*@TpTAP exhibited a significantly larger water contact angle than *E. coli*@TpPa, implying stronger hydrophobic interaction (Figure 3e). This enhanced hydrophobicity facilitated the enrichment of hydrophobic substrates around the cell surface, thereby contributing to the improved catalytic activity. Additionally, second derivative FT‐IR analysis showed that *E. coli*@TpBD displayed redshifts in both the amide I band (C═O stretching vibration) from 1639^−^ to 1633 cm^−1^ and amide II band (N─H/C─N vibration) from 1550^−^ to 1548 cm^−1^, suggesting the formation of a robust hydrogen‐bonding network between *E. coli* and TpBD COF (Figure 3f). In contrast, only the amide I band (C═O stretching vibration) in *E. coli*@TpPa and *E. coli*@TpTAP red‐shifted from 1639^−^ to 1637^−^ and 1635 cm^−1^, respectively, implying fewer active sites participating in the formation of hydrogen bonds [55]. Collectively, the enhanced catalytic activity of *E. coli*@COFs was caused by the synergistic effects of pore size, specific surface area, hydrophobicity, and hydrogen‐bonding interactions. Among them, *E. coli*@TpBD‐2 stood out as the most promising candidate for subsequent investigations due to its optimal integration of these structural and interfacial advantages. This multimodal synergy facilitated effective substrate enrichment and mass transport, highlighting the pivotal role of COFs' structural and interfacial properties in boosting biocatalytic performance.

**FIGURE 3:**
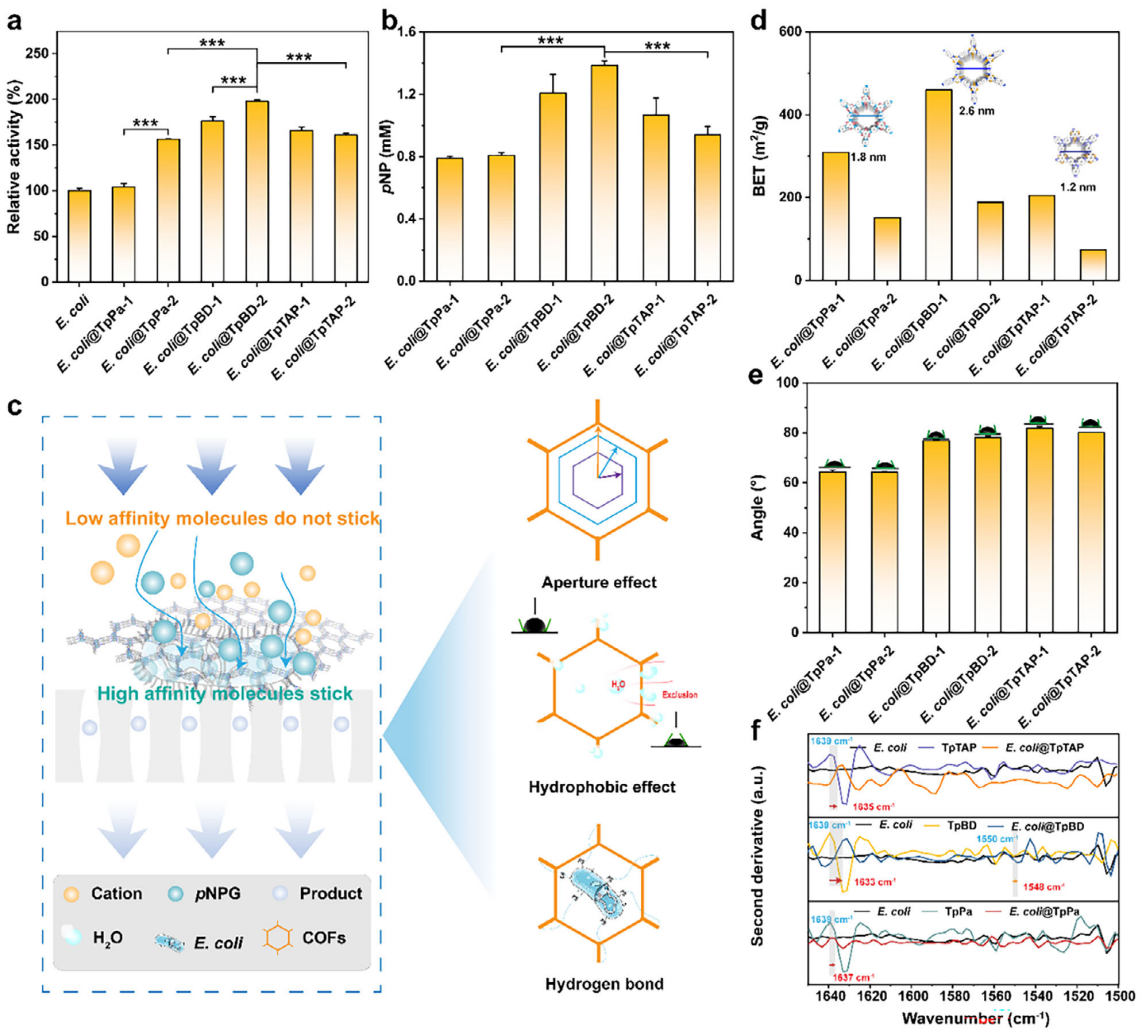
Performance evaluation and mechanism investigation of *E. coli*@COF composites. (a) Comparative catalytic activity of *E. coli* encapsulated by different COF shells. (b) Enrichment capacity analysis of *E. coli*@COF composites. (c) Proposed mechanism for the enhanced catalytic activity of *E. coli*@COF composites. (d) BET surface areas and (e) water contact angles of *E. coli*@COF composites. (f) Second derivatives of FT‐IR spectra of native *E. coli*, pristine COFs, and *E. coli*@COF composites. All data were presented as mean values ± SD of three independent experiments.

### 2.3 Robust Protective Effect of *E. coli*@TpBD‐2 Against Harsh Conditions

In practical biotransformation scenarios, microbial cells are inherently vulnerable to denaturation, activity attenuation, or even lysis when exposed to adverse stimuli [46]. These detrimental factors include temperature/pH fluctuations, toxic organic solvents, enzymes, and UV irradiation (Figure 4a), all of which severely undermine stability and reusability in industrial or complex reaction systems [56]. Consequently, we systematically evaluated the protective efficacy of the COF shell in shielding encapsulated cells against these harsh conditions. As shown in Figures S25 and S26, the residual activities of both uncoated *E. coli* and *E. coli*@TpBD‐2 gradually decreased with prolonged exposure to UV irradiation or high‐temperature treatment. Notably, *E. coli*@TpBD‐2 maintained higher residual activity than uncoated *E. coli* under these conditions. Specifically, the uncoated *E. coli* exhibited a sharp decline in catalytic activity under UV irradiation, retaining only 44.6% and 24.0% of its initial activity under 4 W and 8 W conditions, respectively. In contrast, *E. coli*@TpBD‐2 maintained 60.8% and 53.2% of its activity under the same UV treatment conditions (Figure 4b). Similarly, after the treatment at 50°C, 70°C and 90°C for 3 h, the residual activities of uncoated *E. coli* decreased to 64.0%, 48.0% and 5.7%, whereas *E. coli*@TpBD‐2 preserved 85.0%, 52.5% and 13.7% of its initial activity under these conditions (Figure 4c). This superior protective performance was attributed to the dense pore structure of TpBD‐2, which physically blocked UV radiation. Additionally, the material's low thermal conductivity enabled the formation of a physical insulating layer, which mitigated heat transfer to the encapsulated cells.

**FIGURE 4:**
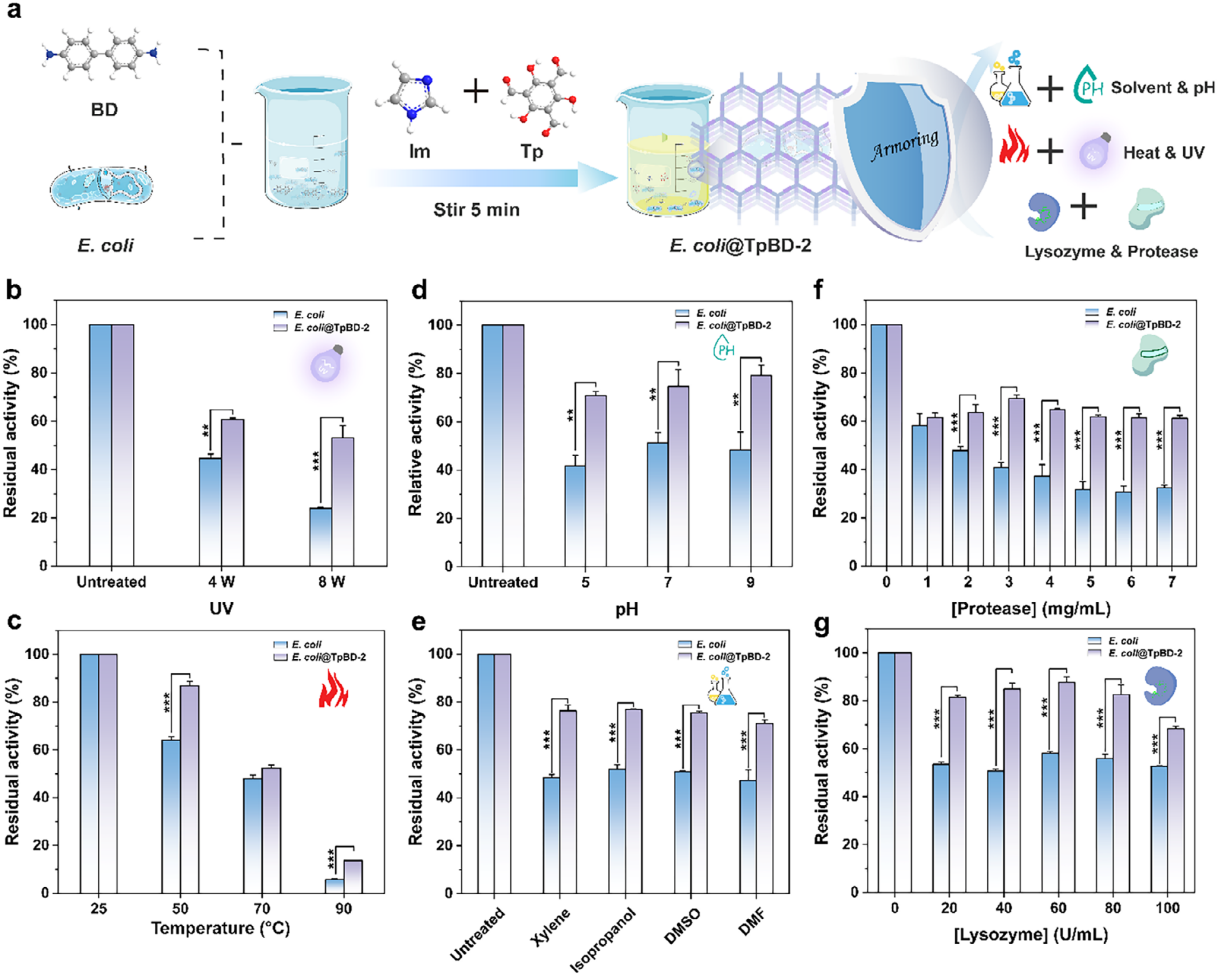
Environmental tolerance evaluation of *E. coli*@TpBD‐2. (a) Schematic diagrams highlighting the protective role of the TpBD‐2 shell in safeguarding *E. coli* against various environmental stressors. Tolerance profiles of native *E. coli* and *E. coli*@TpBD‐2 under different harsh conditions. (b) 1 h of UV irradiation, (c) 3 h of thermal treatment at different temperatures, (d) 8 h of incubation under different pH conditions, (e) 1 h of exposure to different organic solvents, (f) 6 h of incubation with protease solutions of different concentrations, and (g) 6 h of incubation with lysozyme solutions of different concentrations. All data were presented as mean values ± SD of three independent experiments.

Further, we assessed the stability of *E. coli* and *E. coli*@TpBD‐2 under pH fluctuations and exposure of organic solvents. Under the prolonged stress, although both *E. coli* and *E. coli*@TpBD‐2 showed a gradual decline in activity over time, *E. coli*@TpBD‐2 consistently outperformed the uncoated *E. coli* in the retention of residual activity (Figure S27). After 8 h of pH stress (pH 5.0, 7.0, and 9.0), the uncoated *E. coli* underwent a steep drop of activity (to below 52%), while *E. coli*@TpBD‐2 retained over 70% of its activity (Figure 4d). This phenomenon was caused by the interactions between TpBD‐2 and the cell surface, which stabilized the phospholipid bilayer structure of the cell membrane. This stabilization mitigated the abnormal membrane fluidity induced by pH fluctuations, thereby preventing the disturbance of intracellular electrolyte balance [57]. Moreover, after 1 h of exposure to different organic solvents (xylene, isopropanol, DMSO, and DMF), the catalytic activity of uncoated *E. coli* dropped to below 52%, while *E. coli*@TpBD‐2 maintained over 70% of its initial activity (Figure 4e and Figure S28). This resistance was ascribed to the solvent‐stable imine bonds in the TpBD‐2 framework, which formed a physical barrier that impeded the penetration of organic solvents into the encapsulated cells [58].

Protease and lysozyme are common deleterious factors in industrial biocatalysis, as they induce cell lysis and intracellular enzymatic degradation, thereby impairing catalytic efficiency. Therefore, we further explored the stability of *E. coli* and *E. coli*@TpBD‐2 in the presence of these enzymes. After exposing *E. coli* and *E. coli*@TpBD‐2 to protease solutions of different concentrations for 6 h, the activity of *E. coli*@TpBD‐2 remained above 61.0%, which was significantly higher than that of uncoated *E. coli* (Figure 4f,g, and Figure S29). Moreover, after 6 h of exposure to a 100 U/mL lysozyme solution, *E. coli*@TpBD‐2 retained 68.4% of its activity, whereas the activity of *E. coli* declined to 52.7%. These results were attributed to the pore size of TpBD‐2 (2.6 nm), which was significantly smaller than those of the protease (4.2 × 4.5 × 4.4 nm) and lysozyme (4.5 × 3.0 × 3.0 nm) [59, 60]. This structural feature enabled a size‐screening effect that physically blocked the harmful macromolecules from contacting the cells, thereby minimizing cell lysis and intracellular enzymatic degradation [61]. Further, TpBD‐2 showed no identifiable changes in crystal structure upon exposure to high temperatures, varying pH values, organic solvents, and enzymatic treatments (Figure S30), which underscored the structural robustness of the COF shell. This inherent stability was critical for sustaining its protective functionality, as it prevented the efficacy loss caused by self‐degradation under harsh stress conditions. Taken together, the TpBD‐2 COF shell engineered on the surface of *E. coli* substantially improved the bacterium's environmental adaptability and the stability of its intracellular enzymatic activity. This improvement was achieved via the synergistic effects of physical barrier, chemical stability, and size selectivity, providing experimental support for its potential applications under harsh industrial settings.

### 2.4 Continuous‐Flow Reaction for the Production of Rare Ginsenoside CK

Harnessing the enhanced biocomponent stability, processability, and operational condition tolerance conferred by the TpBD‐2 COF shell, we further developed a reactor to evaluate its catalytic performance in a continuous‐flow reaction. This system enabled controlled substrate delivery, enhanced mass transfer, and sustained the enzymatic activity, thereby rendering it a more advantageous approach for industrial‐scale manufacturing [62]. *E. coli* and *E. coli*@TpBD‐2 expressing Ssbgl were packed into a column. A ginsenoside Rb1 solution was pumped into the column inlet at a flow rate of 0.1 mL/min, and the product‐containing effluent was continuously collected for high‐performance liquid chromatography (HPLC) analysis (Figure 5a). Eluted fractions were analyzed by mass spectrometry to verify the biocatalytic transformation of ginsenoside Rb1 to CK. As shown in Figure S31a, Rb1 exhibited a characteristic ion signal at *m/z* 1109.6127, which was consistent with its theoretical value of 1109.29. For the biocatalytic product, two dominant ion signals were detected in positive ion mode, assigned to the sodium adduct ion [M+Na]^+^ at m/z 645.435 and the protonated molecular ion [M+H]^+^ at *m/z* 623.452 (Figure S31b). The detection of CK ion peaks directly confirmed the effective biotransformation from Rb1 to the target rare ginsenoside CK. At 50°C, the space‐time yield (STY) of rare ginsenoside CK catalyzed by *E. coli* reached 0.36 g·L^−1^·d^−1^. Notably, the TpBD‐2 encapsulation endowed the biocatalytic system with excellent stability, maintaining a high conversion rate (consistently above 70%) over an extended reaction period (Figure S32). The optimal temperature for Ssbgl is 90°C, a value substantially higher than the initially adopted 50°C. We therefore adjusted the reaction temperature to balance the catalytic efficiency and system stability, aiming to optimize the CK production. Compared to the reaction conducted at 50°C, *E. coli*@TpBD‐2 exhibited a 1.31‐fold increase in STY at 70°C, reaching 0.67 g·L^−1^·d^−1^. Further, after 10 h of reaction, the conversion rate of *E. coli* dropped to 16.0%. In contrast, the conversion rate of *E. coli*@TpBD‐2 remained at 75.2% (Figure 5b), which further confirmed its excellent stability. Collectively, these results demonstrated that the porous TpBD‐2 shell endowed *E. coli*@TpBD‐2 with exceptional catalytic activity and stability during continuous operation. This advantage originated from the TpBD shell's capacity for substrate enrichment, product separation, and selective interaction with the surrounding environment. Although the immobilized *E. col*i platform enables efficient biosynthesis of rare ginsenosides at the laboratory scale, several technical bottlenecks hinder its large‐scale industrial application. The mass transfer resistance within bioreactors and the product inhibition could reduce the reusability of immobilized cells, and meanwhile, the difficult separation of hydrophobic rare ginsenosides limits the scaled‐up operation. Future work will focus on the optimization of COF materials to enhance the mass transfer and the integration of in situ product extraction to mitigate the inhibition on the reaction, thereby facilitating the industrialization of this biocatalytic system.

**FIGURE 5:**
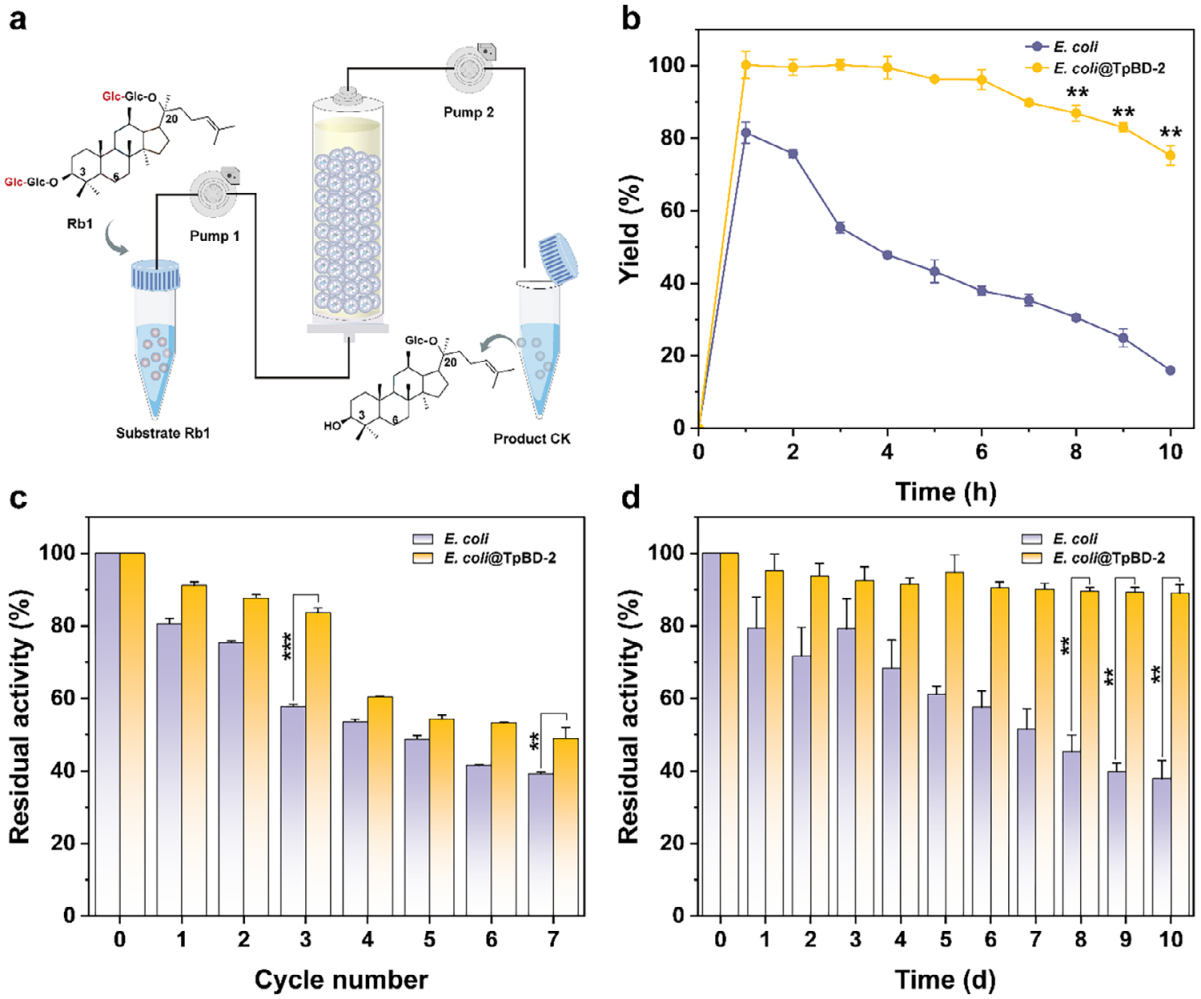
Continuous‐flow synthesis of rare ginsenoside CK from ginsenoside Rb1 and performance analysis of *E. coli*@TpBD‐2 catalyst. (a) Schematic diagram of the continuous‐flow reaction system for the production of ginsenoside CK from ginsenoside Rb1 using *E. coli*@TpBD‐2 as biocatalyst. (b) Time‐dependent content of ginsenoside CK in the continuous‐flow system operated at 70°C with a flow rate of 0.1 mL·min^−1^. (c) Reusability of native *E. coli* and *E. coli*@TpBD‐2 over seven consecutive cycles. (d) Storage stability of native *E. coli* and *E. coli*@TpBD‐2 during the storage at 4°C for different durations. All data were presented as mean values ± SD of three independent experiments.

Moreover, the micron‐scale dimension of these whole‐cell catalysts confers a distinct advantage in recyclability, as they can be efficiently recovered and reused via conventional separation techniques, including centrifugation and filtration. As illustrated in Figure 5c, the intracellular enzymatic activity of uncoated *E. coli* retained only 57.7% of its initial value after three reuse cycles, with a further decline to 39.2% after seven cycles. This pronounced activity loss was attributed to the direct exposure of free *E. coli* to the reaction milieu, which rendered the cells susceptible to environmental stressors and consequent functional degradation. In stark contrast, *E. coli*@TpBD‐2 maintained over 48.9% of its initial intracellular enzymatic activity even after seven consecutive cycles. The potential enzyme leakage from *E. coli*@TpBD‐2 was quantified by assaying the activity of supernatants harvested after each cycle. As shown in Figure S33, only trace activity could be detected across seven cycles, confirming negligible leaching of intracellular enzymes from the composite. This finding demonstrated that the COF shell effectively entrapped the whole cells and intracellular enzymes, which endowed the composite with favorable operational stability during repeated reuse. The regrowth capacity of recovered *E. coli*@TpBD‐2 was assessed to investigate the bacterial physiological status after prolonged continuous‐flow catalysis. As shown in Figure S34, the recycled bacteria exhibited typical growth kinetics featuring an extended lag phase within the first 10 h, followed by rapid exponential proliferation and a stationary plateau after 18 h. These results indicated the outstanding survival stability of whole‐cell biocatalysts under continuous‐flow operation. To elucidate the structural integrity of COF shell post‐recycling, PXRD analysis was performed on *E. coli*@TpBD‐2 after seven cycles. As shown in Figure S35, the PXRD pattern was identical to that of pristine *E. coli*@TpBD‐2, confirming that the COF retained its crystallinity, regular porous architecture, and structural stability throughout the recycling process. These results collectively demonstrated that the TpBD‐2‐mediated whole‐cell immobilization was a viable strategy to enhance the recyclability of *E. coli*‐based catalysts, a key prerequisite for their industrial implementation. Beyond recyclability, superior storage stability is critical for the practical translation of whole‐cell catalytic systems, as it directly governs their feasibility for large‐scale deployment and long‐term operational reliability in industrial settings. As depicted in Figure 5d, *E. coli*@TpBD‐2 retained 89.0% of its initial activity after 10 days of storage at 4°C, whereas the activity of unmodified *E. coli* plummeted to 37.8% under the same conditions. In addition, after 9 days of storage at 25°C, the activity of *E. coli* exhibited a near‐complete loss of activity, while *E. coli*@TpBD‐2 remained 37.2% of its initial activity (Figure S36). The exceptional storage stability of *E. coli*@TpBD‐2 was also ascribed to the unique protective mechanism of the porous TpBD‐2 shell, which physically isolated the encapsulated cells from deleterious environmental factors. This critical factor endowed the whole‐cell catalysts with robust adaptability to discontinuous production processes and on‐site catalytic requirements, thereby effectively bridging the technological gap between laboratory‐scale investigations and large‐scale industrial implementation.

## 3 Conclusion

In conclusion, we developed a scalable whole‐cell immobilization platform by in situ assembly of *E. coli* with three COFs (TpPa, TpBD and TpTAP) under mild aqueous conditions. The *E. coli*@COF composites, especially *E. coli*@TpBD‐2, exhibited a significant enhancement in catalytic efficiency, reaching 1.98‐fold higher than that of free *E. coli*. This improvement was attributed to the synergistic effects of COFs, as tunable mesopores enabled rapid substrate/product diffusion, a large specific surface area with hydrophobicity promoted the substrate enrichment, and hydrogen bonding reinforced the *E. coli*‐substrate interactions to enhance reaction dynamics. Moreover, this platform showed excellent universality, enabling the encapsulation of diverse microbes spanning prokaryotic and eukaryotic systems. More importantly, the COF shell improved the tolerance to harsh conditions via the synergistic effects of physical barrier, chemical stability, and size selectivity. Further, the scale‐up synthesis was achieved in a continuous‐flow reactor, where STY of rare ginsenoside CK reached 0.67 g·L^−1^·d^−1^ at 70°C, with 75.2% of the initial conversion rate retained even after 10 h of continuous operation. This study innovated a high‐performance whole‐cell immobilization platform, advancing the commercialization of the biosynthesis of high‐value natural products via whole‐cell catalysis and paving the way for process intensification in biocatalysis.

## Author Contributions

**Ziye Xing**: methodology, investigation, visualization, formal analysis, data curation. **Fengmei Liu**: writing – original draft, methodology, software, data curation, validation, investigation, visualization, formal analysis. **Hongming He**: conceptualization, writing – review and editing, project administration, supervision. **Shan Zhang**: investigation, writing – original draft, visualization, methodology, validation, software, formal analysis, data curation. **Quanshun Li**: conceptualization, resources, supervision, writing – review and editing, funding acquisition. **Xiao Liang**: conceptualization, writing – original draft, funding acquisition, project administration, resources, supervision. **Shanrong Wei**: investigation, visualization, formal analysis, resources, methodology.

## Funding

The work was supported by the National Natural Science Foundation of China (U24A20365, 32301051 and 32271319), the Science and Technology Department of Jilin Province (20250205039GH), the Development and Reform Commission of Jilin Province (2023C015), the China Postdoctoral Science Foundation (2025T180729) and the Fundamental Research Funds of the Central Universities, China (2024‐JCXK‐11).

## Declaration of Generative AI and AI‐Assisted Technologies in the Writing Process

During the manuscript preparation, AI tools (ChatGPT) were only used to improve grammar, sentence structure and readability. All the contents have been carefully checked, edited and approved by the authors to ensure scientific accuracy.

## Conflicts of Interest

The authors declare no conflicts of interest.

## Supporting information




**Supporting File**: advs77136‐sup‐0001‐SuppMat.docx.

## Data Availability

The data that support the findings of this study are available from the corresponding author upon reasonable request.
