# Supplementary material for: Tailored Covalent Organic Frameworks Enable the Encapsulation of Living Cells to Produce Rare Ginsenoside
Source: Adv Sci (Weinh). 2026 Aug 13;13(61):e77136. doi: 10.1002/advs.77136 (PMC13729983; doi:10.1002/advs.77136)
Supplement: Supplementary file 1 [file ADVS-13-e77136-s001.docx]

Supporting Information

Tailored Covalent Organic Frameworks Enable the Encapsulation of Living Cells to Produce Rare Ginsenoside

Fengmei Liu,^+[a]^ Shan Zhang,^+[b]^ Shanrong Wei,^[a]^ Ziye Xing,^[a]^ Hongming He,^*[b]^ Xiao Liang,^*[a]^ and Quanshun Li^*[a]^

**Materials and Methods**

**Materials**

Phenylenediamine (Pa, 99%), benzidine (BD, 98%), 1,3,5-triformylphloroglucinol (Tp, 98%), and glutaraldehyde (GA, 98%) were acquired from Macklin (Shanghai, China). Isopropyl β-D-1-thiogalactopyranoside (IPTG, ≥99%, ≤0.1% dioxane) and ethylenediaminetetraacetic acid (EDTA, ≥99%) were purchased from Sigma (Shanghai, China). 2,4,6-Tris(4-aminophenyl)-1,3,5-triazine (TAPT, 98%) was obtained from Heowns (Tianjin, China). Methanol (MeOH, AR), acetonitrile (ACN, 99%), xylene (AR), *N,N*-dimethylformamide (DMF, AR), dimethyl sulfoxide (DMSO, AR), isopropanol (AR), and tert-butanol were acquired from Fuyu Fine Chemical (Tianjin, China). Sodium chloride (NaCl, 99.5%), potassium dihydrogen phosphate (KH_2_PO_4_, 99%), dipotassium hydrogen phosphate (K_2_HPO_4_, 99%), cetyltrimethylammonium bromide (CTAB, 99%), and imidazole (99.5%) were provided by Aladdin (Shanghai, China). The bacterial live/dead staining kit was obtained from Beyotime (Jiangsu, China). Protease, lysozyme, ginsenoside Rb1 (98%), ginsenoside CK (98%), and *p*-nitrophenyl-β-D-glucopyranoside (*p*NPG, 98%) were obtained from Yuanye (Shanghai, China). Tween 80 and Rhodamine B (RhB, ≥98%) were purchased from Solarbio (Beijing, China). All the reagents were used as received without further purification.

**Characterization**

Transmission electron microscopy (TEM) images were obtained using a Tecnai G2 F20 electron microscope (FEI, America). Field emission scanning electron microscopy (FE-SEM) images were obtained on a Hitachi SU-8010 microscope (Tokyo, Japan), operated at 5 kV. Powder X-ray diffraction (PXRD) pattern was carried out on an X-ray diffractometer (DX-2700BH, Dandong Haoyuan, Liaoning, China). Fourier transform infrared (FT-IR) spectra within the wavenumber range of 4000-400 cm^-1^ were recorded on Bruker Alpha spectrometer (Billerica, MA, USA). N_2_ sorption isotherms were detected using a surface area and pore size analyzer (Micromeritics ASAP 2020 HD88, USA). Solid-state nuclear magnetic resonance (NMR) spectra were obtained on a Bruker Avance III HD 400 MHz spectrometer (Billerica, MA, USA). Ultraviolet-visible (UV-Vis) spectra were performed on a UV-2700i spectrophotometer (Shimadzu, Tokyo, Japan) using a 1-cm path length quartz cuvette. The static water contact angle measurement was conducted using a JY-82C video contact angle meter (Dingsheng, Chengde, China) at room temperature. Confocal laser scanning microscopy (CLSM) was performed on a LSM 900 (Carl Zeiss, Gottingen, Germany) by using argon laser with an excitation and emission filter of 488 nm and 525 nm, respectively.

**Sample Preparation for SEM and TEM**

Briefly, *Escherichia coli* (*E. coli*) cells were first resuspended in a 2.5% (v/v) glutaraldehyde solution and statically incubated at 4 °C overnight for fixation. Fixed cells were harvested by centrifugation at 8000× *g*, followed by three sequential washes with 0.2 M phosphate-buffered saline (PBS, pH 6.8). Post-washing, the samples were dehydrated through a graded ethanol series (30%, 50%, 70%, 80%, 90%, and 100%, v/v), with 15 min of immersion at each concentration. Subsequently, the dehydrated cells were transferred to an ethanol: *tert*-butanol mixture (1:1, v/v) and incubated for 15 min. After the centrifugation at 8000× *g* to remove the supernatant, the samples underwent two rounds of 100% *tert*-butanol replacement (15 min per round) to eliminate residual ethanol. For SEM sample preparation, the dehydrated samples were carefully transferred to a clean silicon wafer to optimally preserve the morphology during drying, followed by gold sputter-coating to enhance conductivity. For TEM sample preparation, a small volume of the dehydrated sample suspension was pipetted onto a 300-mesh carbon-coated copper grid. Excess suspension was gently blotted with filter paper, and the copper grid was placed in a cleanroom environment for complete air-drying at room temperature.

**Preparation of Engineered Strain for Ssbgl Expression**

The gene encoding *Sulfolobus solfataricus* β-glycosidase (Ssbgl; Genbank Accession No.: WP_009992676) was custom-synthesized by GenScript (Nanjing, Jiangsu, China) with codon optimization for heterologous expression in *E. coli*. The optimized gene was cloned into pET-24a(+) vector *via* restriction digestion-ligation to construct recombinant plasmid pET-24a-Ssbgl. This recombinant plasmid was then transformed into competent *E. coli* BL21(DE3) cells. The recombinant strain was inoculated into LB medium supplemented with 50 μg/mL kanamycin and cultured at 37 °C with shaking at 180 rpm. Cell density was monitored by measuring the optical density at 600 nm (OD_600_) using a UV-Vis spectrophotometer. When the culture reached an OD_600_ of 0.6, filter-sterilized IPTG stock solution was added to a final concentration of 0.5 mM to induce the soluble expression of Ssbgl protein. To ensure sufficient enzyme accumulation while reducing insoluble inclusion bodies, the culture was further incubated at 20 °C overnight with constant shaking at 180 rpm, which facilitated slow folding of Ssbgl and suppressed the formation of protein aggregates. Bacterial cells were subsequently harvested by the centrifugation at 4 °C and 4000× g for 15 min.

**Enzymatic Activity Assay of Ssbgl**

The catalytic activities of *E. coli* (harboring recombinant Ssbgl) and *E. coli*@COFs were evaluated by quantifying the production of *p*-nitrophenol (*p*NP), a hydrolysis product of *p*NPG. The catalytic reaction system (total volume: 800 µL) consisted of 360 µL of phosphate buffer (50 mM, pH 7.0), 400 µL of *p*NPG solution (5 mM), and 40 µL catalyst. Two catalyst groups were tested: (1) *E. coli* suspension (0.4 mg wet cell weight, with a cell density of 1.26×10^9^ CFU/mg); (2) *E. coli*@COFs, loaded with an equivalent amount of *E. coli* cells to ensure equivalent catalyst dosage across groups. The wet weight of *E. coli* cells was determined by weighing the cell pellet harvested *via* the centrifugation at 4000× g for 15 min. The catalytic reaction was monitored by measuring the absorbance increase at 405 nm using a UV-Vis spectrophotometer. The optimal temperature for *E. coli* (recombinant Ssbgl-expressing *E. coli*) was determined by measuring its catalytic activity at pH 7.0 (50 mM phosphate buffer) over a temperature range of 50-100 °C. Similarly, the optimal pH was evaluated by assaying the catalytic activity at 25 °C across a pH range of 5.0-9.0 (buffers adjusted to respective pH values as needed). For both assays, the maximum enzymatic activity detected under the tested conditions was defined as 100%, with all other activity values normalized to this reference. All experiments were performed in triplicate, and the results are presented as mean values ± standard deviation (SD).

**Cell Permeabilization**

Bacterial cells were collected by the centrifugation at 4000× g for 15 min at 4 °C and resuspended in 2 mL of phosphate buffer (50 mM, pH 7.0). Two permeabilization strategies were performed. For chemical treatment, the cell suspension was treated with EDTA, methanol, or NaCl (final concentrations as specified in the experimental design) for 30 min each. For physical treatment, the cells were exposed to low temperatures (-20 °C, -80 °C, or -160 °C) for 30 min each. Following the permeabilization, the treated cells were immediately subjected to the catalytic activity assay of Ssbgl using the established protocol.

**Synthesis of TpPa-COF**

For the standard synthesis of TpPa-COF, two distinct monomer feed ratios were adopted to prepare COFs with different thicknesses: 0.06/0.04 mmol (6.6 mg Pa + 8.4 mg Tp) and 0.03/0.02 mmol (3.3 mg Pa + 4.2 mg Tp). Specifically, the respective amounts of Pa and Tp for each feed ratio were placed into individual 100 mL vials. To each vial, 10 mL of PBS was added, and the mixtures were sonicated until the monomers were completely dissolved. Imidazole was subsequently added at dosages matching the monomer feeds: 200 mg for the higher monomer feed and 100 mg for the lower one. The mixtures were stirred for 5 min to initiate the reaction, and the resulting TpPa-COF solids were collected *via* sequential filtration followed by centrifugation at 8000g for 5 min.

**Scheme S1**. Schematic illustration of the synthesis reaction for TpPa-COF.

**Synthesis of TpBD-COF**

For the standard synthesis of TpBD-COF, two different monomer feed ratios (BD/Tp) were employed to prepare COFs with varying thicknesses: 0.06/0.04 mmol (11.0 mg BD and 8.4 mg Tp) and 0.03/0.02 mmol (5.5 mg BD and 4.2 mg Tp). Specifically, the respective amounts of BD (diamine monomer) and Tp (aldehyde monomer) for each feed ratio were placed into 100-mL vials. For TpBD-COF synthesis, subsequent procedures followed those for TpPa-COF. Specifically, 10 mL of PBS was added to each vial, and the mixtures were sonicated until the monomers were completely dissolved. Imidazole was then added at dosages matching the monomer feeds, and the mixtures were stirred for 5 min to initiate the imine polymerization. Finally, the TpBD-COF solids were collected via sequential filtration and centrifugation at 8000g for 5 min.

**Scheme S2**. Schematic illustration of the synthesis reaction for TpBD-COF.

**Synthesis of TpTAP-COF**

For standard synthesis of TpTAP-COF, two TAPT/Tp monomer feed ratios were employed to prepare COFs with different thicknesses. The ratios were 0.04/0.04 mmol (14.0 mg TAPT + 8.4 mg Tp) and 0.02/0.02 mmol (7.0 mg TAPT + 4.2 mg Tp). Specifically, TAPT (triamine monomer) and Tp (aldehyde monomer) at each feed ratio were placed into individual 100-mL vials, with subsequent synthesis procedures identical to those described for TpPa-COF.

**Scheme S3**. Schematic illustration of the synthesis reaction for TpTAP-COF.

**Large-Scale Synthesis of TpBD-COF**

For the gram-scale synthesis of TpBD-COF, BD (13.83 g, 75 mmol) was transferred to a 1-L glass reaction vessel. PBS solution (500 mL) was introduced, and the suspension was subjected to the sonication until BD was completely dissolved. Tp (10.5 g, 50 mmol) and imidazole (3.333 g, 50 mmol) were sequentially added in the sample. The mixture was stirred at room temperature for 2 h to initiate the imine polymerization. The precipitate was isolated, sequentially rinsed with DMF, deionized water and methanol. After the vacumm drying at 80 °C overnight, solid TpBD-COF was collected (18.6 g). Comparative characterizations of lab-scale and gram-scale TpBD-COF samples were performed to benchmark the scalability of this synthetic route.

**Preparation of *E. coli*@COFs**

In a typical preparation, a defined amount of Pa, BD, or TAPT was pre-added separately to 100 mL glass vials. Each amino monomer corresponded to an independent *E. coli*@COF experimental system, matching the monomer types used in the synthesis of TpPa-COF, TpBD-COF and TpTAP-COF. The specific amounts were 0.06/0.03 mmol Pa, 0.06/0.03 mmol BD, and 0.04/0.02 mmol TAPT. Subsequently, 10 mL of PBS was added to each vial, and the systems were sonicated until the organic monomers were completely dissolved. After dissolution, 0.16 g of wet *E. coli* cells was added to each system and uniformly dispersed by stirring. All the cell pellets were harvested from a single batch under synchronized cultivation and induction conditions to minimize the variability in intracellular enzyme expression across all the samples. This step was followed by the addition of Tp (8.4 mg for the higher monomer feed, 4.2 mg for the lower feed) and imidazole (200 mg for the higher feed, 100 mg for the lower feed), at dosages consistent with the TpPa-COF synthesis. The mixtures were stirred for 5 min to initiate the imine polymerization and *E. coli*@COF composite formation, and the resulting *E. coli*@COF solids were collected by sequential filtration and centrifugation at 8000g for 5 min. Statistical quantification of COF shell thickness distribution was performed on TEM images. Twenty cells were randomly selected, with three independent detection regions analyzed for each cell. All the shell thickness values were measured *by* the software ImageJ, and the average measurements were used to characterize the overall COF shell thickness distribution.

**CLSM Analysis**

Briefly, *E. coli*, *Bacillus 168*, *S. aureus*, *E. coli Nissle*, *M. smegmatis*, and *P. pastoris* were all cultured to the early logarithmic phase (OD_600_ ≈ 0.6). These cells were collected by centrifugation (4000× g, 5 min, 4 °C) and resuspended in 2 mL of PBS (50 mM, pH 7.0) to a final concentration of 10^8^ cells/mL. Subsequently, 200 μL of rhodamine B solution (1 mg/mL, dissolved in ultrapure water) was added to the cell suspension. The mixture was stirred at 4 °C for 12 h in the dark to ensure sufficient dye binding to the cell surface. After the incubation, the mixture was centrifuged (4000× g for 5 min, 4 °C) to collect the cells. The isolated cell pellet was washed three times with 70% (v/v) ethanol (to remove the unbound rhodamine B) and then once with PBS to neutralize residual ethanol, yielding rhodamine B-labeled cells. Fluorescence imaging of the labeled cells was performed using a Zeiss LSM900 microscope (Oberkochen, Germany). Rhodamine B was excited using a 543 nm He-Ne laser, and the emission signal was collected through a 560 nm band-pass filter. The immobilization procedure for rhodamine B-labeled cells was identical to that of native cells, except that an equal amount of rhodamine B-labeled cells was used instead of native cells.

**Live/Dead Bacterial Staining Assay**

First, native *E. coli* cells and *E. coli*@COFs were suspended in physiological saline and adjusted to a final concentration of 10^8^ cells/mL. Subsequently, *N*,*N*-dimethylaniline N-oxide (DMAO, a live-cell fluorescent dye) and propidium iodide (PI, a dead-cell fluorescent dye) were prepared into a staining working solution following the instructions of the corresponding assay kits. This working solution was then added to the cell suspension at a volume ratio of 1:100 (working solution: cell suspension), and the mixture was incubated at 37 °C in the dark for 15 min to ensure sufficient dye-cell binding. The treated cells were washed three times with physiological saline to remove the unbound dye. Fluorescence images were captured using an Olympus IX71 fluorescence microscopy (Tokyo, Japan) with a 20× objective lens. The excitation/emission (Ex/Em) wavelengths were set to 503/530 nm for DMAO and 535/617 nm for PI, respectively. In addition, microbial viability was quantified *via* dual DMAO/PI staining combined with a multi-mode microplate reader. Briefly, *E. coli* was cultured in LB medium at 37 °C until the mid-logarithmic growth. The cells were harvested by centrifugation and resuspended in sterile 0.9% NaCl solution. The bacterial suspension was adjusted to 10^8^ CFU/mL and further diluted 100-fold with PBS to a final concentration of 10^6^ CFU/mL. 100 μL of diluted cell suspension was transferred to a 96-well plate and supplemented with 1 μL of 100× staining working solution. The mixture was incubated at 37 °C in the dark for 30 min prior to the fluorescence measurement using SpectraMax iD3 multi-mode microplate reader (Molecular Devices, USA).

**Determination of Cell Growth Curve of *E. coli*@COFs**

Briefly, equal amounts of native *E. coli* cells and *E. coli*@COFs were separately suspended in 5 mL of LB medium supplemented with 50 μg/mL kanamycin. The suspensions were then inoculated and cultured in a shaking incubator at 37 °C and 180 rpm. Once the cultures reached the early logarithmic phase (OD_600_ ≈ 0.3), the OD_600_ values of the cell suspensions were recorded every 1 h over 21-h period using a UV-Vis spectrophotometer to generate the cell growth curves. All experiments were conducted in biological triplicate to ensure the data reliability.

**Assessment of Substrate Enrichment by COF Shells**

*E. coli*@COFs were prepared using *E. coli* without exogenous genes. Equal volumes of the prepared *E. coli*@COFs and the corresponding native *E. coli* were separately mixed with 2 mM *p*NPG, followed by the incubation for 1 h with gentle shaking. The mixture was then centrifuged at 12,000 × g for 5 min, and the *p*NPG concentration in the supernatant was quantified using a UV-Vis spectrophotometer.

**Adsorption Kinetic Experiment**

For the adsorption kinetic measurements, 10 mg of *E. coli*@COFs was dispersed in 5 mM *p*NP solution. The resulting suspension was incubated at room temperature with continuous shaking at 120 rpm over a series of time intervals. After the sedimentation, supernatants were harvested for UV-Vis detection. The *p*NP adsorption capacity was calculated based on the standard calibration curve recorded at 405 nm. Nonlinear fitting via the pseudo-second order equation was adopted to process the acquired kinetic data, and the equation is expressed as:

$$qt=\frac{k_{2}q_{e}^{2}t}{1+k_{2}q_{e}t}$$

where *q_t_* (mg/g) refers to the adsorption capacity at time t, *q_e_* (mg/g) is the equilibrium adsorption capacity, *k_2_* denotes the pseudo-second-order kinetic rate constant, and *t* (min) corresponds to the adsorption time.

**Evaluation of Protective Effect against External Stresses**

To evaluate the protective effect of COF coating on *E. coli*, native *E. coli* cells and *E. coli*@COFs were subjected to a series of stress conditions, and their residual catalytic activity was determined thereafter. For biphasic water-organic system stress, the suspensions were incubated in water-organic mixtures (volume ratio 4:1) containing isopropanol, xylene, DMF, or DMSO at 37 °C and 180 rpm for varied durations. For pH stress, samples were resuspended in buffer solutions with different pH values (pH 5.0: acetate buffer; pH 7.0: PBS; pH 9.0: Tris-HCl buffer) and incubated at 37 °C for varied durations. For UV irradiation stress, cell suspensions were exposed to UV (254 nm) irradiation at 4 W or 8 W (distance: 15 cm), respectively, for varied durations. For thermal stress, cell samples were incubated at 50 °C, 70 °C, or 90 °C in a water bath for varied durations. For enzymatic treatment stress, protease or lysozyme (final concentrations adjusted per experimental design) was added to the cell suspensions and incubated at 37 °C for different time. Following the treatment, the samples were collected by centrifugation (4000× g, 5 min), washed three times with PBS, and subjected to the measurement of residual activity. All experiments were performed in biological triplicate.

**Storage Stability of *E. coli*@COFs**

To assess the storage stability of native *E. coli* cells and *E. coli*@COFs, the following experiment was performed. Equal aliquots of the two samples (adjusted to 10^8^ cells/mL with 50 mM PBS, pH 7.0) were separately stored in sealed sterile centrifuge tubes at 4 °C and 25 °C, respectively. Over an 11-day storage period, samples were periodically withdrawn at predetermined time points, and the residual catalytic activity of Ssbgl was measured using *p*NPG as the substrate. All experiments were conducted in biological triplicate, with the activity on Day 0 defined as 100% for normalization.

**Reusability of *E. coli*@COFs**

To evaluate the reusability of Ssbgl in native *E. coli* cells and *E. coli*@COFs, the following experiment was conducted. Equal aliquots of the two samples (adjusted to 10^8^ cells/mL with PBS, pH 7.0) were separately added to 5-mL reaction system containing 1 mM *p*NPG. Each catalytic reaction was conducted at 37 °C for 30 min (defined as one reaction cycle). After the completion of each cycle, the samples were separated *via* centrifugation at 4000× g for 15 min, followed by washing with PBS three times. The treated samples were then transferred to a fresh 5-mL reaction system (identical to the initial system: 1 mM *p*NPG in PBS, pH 7.0) to initiate the next catalytic cycle. This process was repeated for 7 consecutive cycles, and the residual activity of each cycle was determined using *p*NPG as the substrate, with the activity of the first cycle defined as 100% for normalization. Moreover, an enzyme leakage quantification assay was performed to monitor the enzyme leaching from *E. coli*@TpBD-2 during repeated uses. Upon the completion of each cycle, the supernatant was collected through the centrifugation, and the enzymatic activity was measured using *p*NPG as the substrate. All experiments were performed in biological triplicate.

**Biocatalytic Conversion of Rb to CK in a Continuous-Flow Reactor System**

*E. coli* and *E. coli*@TpBD-2 (both harboring the Ssbgl gene) were packed into a column reactor (internal diameter: 2 cm, volume: 10 mL). A sand filter (pore size: 5-10 μm) was placed at the base of the column, and 300-mesh screens fitted at both ends. The reaction column was maintained at a constant temperature in a water bath. A 0.05 mg/mL of Rb1 solution (prepared with PBS) was pumped into the column reactor at a flow rate of 6.0 mL/h. The Rb1 solution passed through the bottom of the column reactor, exited from the top, and the effluent was collected periodically for high performance liquid chromatography (HPLC) analysis. HPLC analysis was performed on a ShimNex CS system equipped with a UV detector and a C18 chromatographic column (4.6 × 250 mm, 5 μm). The detection was carried out at a wavelength of 203 nm, with the mobile phase consisting of water/acetonitrile (40:60, v/v) containing 0.1% trifluoroacetic acid (TFA). The flow rate was fixed at 1.0 mL/min, column temperature was maintained at 30 °C, and 10 μL sample solution was injected for each test. The chromatographic column was pre-equilibrated with the prepared mobile phase for 30 min prior to the sample injection to obtain a stable baseline. Moreover, ginsenoside Rb1 and its biocatalytic product were analyzed on Waters ACQUITY UPLC I-Class/XEVO G2-XS QTOF-MS system (Waters, Milford, MA, USA). All the samples were diluted in methanol prior to analysis. Electrospray ionization (ESI) was employed, and mass spectra were recorded under both positive and negative ionization modes over an *m/z* range of 100-1500. The capillary voltage was set at 3.0 kV for positive mode and 2.5 kV for negative mode. The cone voltage was held at 40 V, and the desolvation gas flow rate was set as 800 L/h with ion source temperature of 120 °C. Argon was served as the collision gas, with collision energies ranging from 20-50 eV. Real-time calibration of raw data was performed using the instrument’s LockMass channel.

**Regrowth Assay of** ***E. coli*@TpBD-2 after Continuous-Flow Catalysis**

Briefly, *E. coli*@TpBD-2 were harvested from the reactor column after 5 h of catalytic operation. The collected composites were washed with sterile saline and resuspended in fresh LB medium to an initial OD_600_ of 0.05. The culture was incubated at 37 °C under continuous shaking at 180 rpm. At predetermined time intervals, aliquots of bacterial suspension were collected, and OD_600_ value was measured using a UV-Vis spectrophotometer.

**Statistical analysis**

Statistical significance of multiple comparison tests was calculated via one-way analysis of variance (ANOVA) using GraphPad Prism 9.0. The statistical significance was presented as **p* < 0.05, ***p* < 0.01 and ****p* < 0.001.

**Supporting Figures**

**Figure S1**. FT-IR spectra of TpPa-COF synthesized at different reaction time.

**Figure S2.** FT-IR spectra of TpBD-COF synthesized at different reaction time.

**Figure S3.** FT-IR spectra of TpTAP-COF synthesized at different reaction time.

**Figure S4.** (a) SEM image and (b) TEM image of TpPa-COF.

**Figure S5.** (a) SEM image and (b) TEM image of TpBD-COF.

**Figure S6.** (a) SEM image and (b) TEM image of TpTAP-COF.

**Figure S7.** AA-stacked atomic models of (a) TpPa, (b) TpBD and (c) TpTAP. Atoms: gray (C), blue (N), red (O), white (H).

**Figure S8.** (a) N_2_ sorption isotherms, (b) FT-IR spectra, (c) SEM, (d) TEM and (e) HRTEM of the scaled-up TpBD-COF.

**Figure S9.** Schematic representation of the detection of Ssbgl activity in *E. coli* BL21 cells using *p*NPG as the substrate.

**Figure S10.** Effects of (a) EDTA concentration, (b) methanol concentration and (c) NaCl concentration on the catalytic activity of *E. coli*. All data were presented as mean values ± SD of three independent experiments.

**Figure S11.** Effect of permeabilization treatments on the catalytic activity of *E. coli*. All data were presented as mean values ± SD of three independent experiments.

**Figure S12.** SEM images of *E. coli* cells treated with different permeabilization methods.

**Figure S13.** Pore size distribution of *E. coli*@COFs prepared with different COF shells and feeding amounts. (a) *E. coli*@TpPa-1 and *E. coli*@TpPa-2, (b) *E. coli*@TpBD-1 and *E. coli*@TpBD-2, and (c) *E. coli*@TpTAP-1 and *E. coli*@TpTAP-2. -1 denotes high feeding amount, and -2 denotes low feeding amount.

**Figure S14.** SEM images of *E. coli*@COFs prepared with different COF shells and feeding amounts. -1 denotes high feeding amount, and -2 denotes low feeding amount.

**Figure S15.** TEM images of *E. coli*@COFs prepared with different COF shells. The annotated values represent the local COF shell thickness at the marked spots.

**Figure S16.** Statistical distribution of COF shells for six *E. coli*@COF composites. Three local shell thickness regions were measured on each bacterial cell, with a total of 20 cells analyzed for every sample.

**Figure S17.** Live/dead assay images of *E. coli* and *E. coli*@COFs. Green fluorescence indicates live cells, and red fluorescence represents the dead cells.

**Figure S18**. Fluorescence intensities of DMAO and PI for free *E. coli* and *E. coli*@COFs. DMAO and PI signals correspond to viable and dead bacterial cells, respectively.

**Figure S19.** Growth curves of *E. coli* (gray curve) and *E. coli*@COFs (colorful curve) for assessing the biocompatibility of the immobilization process. All data were presented as mean values ± SD of three independent experiments.

**Figure S20.** CLSM images of *E. coli* BL21, *S. aureus*, *Bacillus 168*, *E. coli* Nissle, *M. smegmatis*, and *P. pastoris*. The cells were labeled with Rhodamine B (red).

**Figure S21.** CLSM images of *E. coli* BL21@TpBD-2, *S. aureus*@TpBD-2, *Bacillus* 168@TpBD-2, *E. coli* Nissle@TpBD-2, *M. smegmatis*@TpBD-2 and *P. pastoris*@TpBD-2. The cells were labeled with Rhodamine B (red).

**Figure S22.** (a) PXRD and (b) FT-IR characterization of TpBD-2-encapsulated whole cells of diverse microbial strains.

**Figure S23.** Enzymatic characterization of *E. coli* (blue curve) and Ssbgl protein (yellow curve): (a) optimal temperature and (b) optimal pH. All data were presented as mean values ± SD of three independent experiments.

**Figure S24**. Adsorption kinetics of *p*NP onto various *E. coli*@COF composites, where blank *E. coli* chassis cells without Ssbgl expression were adopted.

**Figure S25.** Radiation tolerance of native *E. coli* and *E. coli*@TpBD-2 over different exposure time: (a) 4 W and (b) 8 W irradiation. All data were presented as mean values ± SD of three independent experiments.

**Figure S26.** Thermal tolerance of *E. coli* and *E. coli*@TpBD-2 over time at different temperatures: (a) 50 °C, (b) 70 °C and (c) 90 °C. All data were presented as mean values ± SD of three independent experiments.

**Figure S27.** pH tolerance of *E. coli* and *E. coli*@TpBD-2 over time at different pH levels: (a) pH 5, (b) pH 7 and (c) pH 9. All data were presented as mean values ± SD of three independent experiments.

**Figure S28.** Organic solvent tolerance of native *E. coli* and *E. coli*@TpBD-2 over different time: (a) DMF, (b) DMSO, (c) xylene and (d) isopropanol. All data were presented as mean values ± SD of three independent experiments.

**Figure S29.** Enzymatic tolerance of *E. coli* and *E. coli*@TpBD-2 over different time: (a) protease and (b) lysozyme. All data were presented as mean values ± SD of three independent experiments.

**Figure S30.** PXRD patterns of *E. coli*@TpBD-2 after different treatment conditions.

**Figure S31.** Mass spectra of (a) ginsenoside Rb1 and (b) its biocatalytic product CK.

**Figure S32.** Time-dependent CK yield of *E. coli* and *E. coli*@TpBD-2 in continuous-flow reaction at 50 °C. All data were presented as mean values ± SD of three independent experiments.

**Figure S33**. Quantification of leaked enzymatic activity over seven consecutive cycles. The initial activity at cycle 0 was set as 100% reference.

**Figure S34**. Growth curve of *E. coli*@TpBD-2 recovered from the continuous-flow reactor after 5 h of catalytic operation.

**Figure S35.** PXRD pattern of *E. coli*@TpBD-2 after seven reaction cycles.

**Figure S36.** Storage stability of *E. coli* and *E. coli*@TpBD-2 at 25 °C. All data were presented as mean values ± SD of three independent experiments.

**Supporting Tables**

**Table S1.** **BET surface area and pore size analysis of different COF materials.**

| **Sample** | **BET (m^2^/g)** | **Pore size (nm)** |
| --- | --- | --- |
| TpPa-1 min | 181.58 | 1.78 |
| TpPa-5 min | 602.87 | 1.81 |
| TpPa-10 min | 614.20 | 1.79 |
| TpBD-1 min | 262.83 | 2.60 |
| TpBD-5 min | 921.74 | 2.60 |
| TpBD-10 min | 979.23 | 2.60 |
| TpTAP-1 min | 81.91 | 1.20 |
| TpTAP-5 min | 329.51 | 1.20 |
| TpTAP-10 min | 338.18 | 1.27 |

**Table S2.** **BET surface area and pore size analysis of different *E. coli*@COFs.**

| **Sample** | **BET (m^2^/g)** | **Pore size (nm)** |
| --- | --- | --- |
| *E. coli*@TpPa-1 | 309.39 | 1.69 |
| *E. coli*@TpPa-2 | 151.71 | 1.86 |
| *E. coli*@TpBD-1 | 459.51 | 2.60 |
| *E. coli*@TpBD-2 | 188.69 | 2.61 |
| *E. coli*@TpTAP-1 | 205.33 | 1.27 |
| *E. coli*@TpTAP-2 | 73.76 | 1.27 |
